# The Natural Disinfectant Role of Essential Oils in Improving Radical Scavenging Activity and Total Phenolic Compounds in Fresh Vegetables

**DOI:** 10.3390/antiox14121458

**Published:** 2025-12-05

**Authors:** Ben Allal Hafsa, Quesada-Granados José Javier, Agil Ahmad, Villalón-Mir Marina

**Affiliations:** 1 Department of Nutrition and Bromatology, School of Pharmacy, University of Granada, 18071 Granada, Spain; hafsaelmsiken@correo.ugr.es (B.A.H.); marinavi@ugr.es (V.-M.M.); 2 Department of Pharmacology and Neurosciences Institute, School of Medicine, University of Granada, 18016 Granada, Spain; aagil@ugr.es

**Keywords:** disinfection of vegetables, antiradical activity, total polyphenolic content, thyme essential oil, peppermint essential oil, bleach solutions, microbial growth

## Abstract

The objective of this study was to evaluate the radical scavenging activity (RSA) and total polyphenolic content (TPC) in petiolate vegetables (baby spinach) and sessile vegetables (*Romaine lettuce*) disinfected with essential oils of thyme and peppermint compared with bleach solutions, a chemical disinfectant widely used in food preparation. The vegetables, obtained from local markets in Granada (Spain), were treated with varying concentrations of essential oils and bleach solutions. Antiradical activity was evaluated using the DPPH radical scavenging method, while total polyphenols were determined using the Folin–Ciocalteu reagent. The results showed that essential oils significantly reduced microbial load, with inverse correlations between radical scavenging activity and microbial load and total phenolic compounds. Bleach solutions, on the other hand, show a strong direct correlation, significantly reducing the microbial load as well as the antiradical activity and total phenolic content. However, this antimicrobial and antioxidant effect depends on the morphological characteristics of the vegetable (stemmed or sessile) and the chemical composition of the essential oil. These results suggest that essential oils may be effective natural alternatives for disinfecting vegetables, as they increase their antiradical activity and polyphenolic content, in contrast to sodium hypochlorite, which affects the functional properties of the product by reducing the RSA and TPC.

## 1. Introduction

The World Health Organisation (WHO) and the Food and Agriculture Organisation of the United Nations (FAO) recommend consuming at least 400 g of fruits and vegetables per day to maintain and ensure good health [1]. Many studies have indicated the need to adopt a healthy lifestyle and diet to strengthen our immune system and preserve our physical and mental health [2].

Although vegetables are generally crucial to health, their consumption is associated with the risk of contamination if they are not properly washed and disinfected. One cause of contamination in these foods is the presence of significant amounts of heavy metals that come mainly from the soil, so it is recommended to wash vegetables to remove potentially toxic elements with low phytoaccessibility, such as Pb, where washing with water can reduce its content by up to 50% [3].

Another important source is biological contamination by bacteria, viruses, fungi, and parasites [3,4,5], with the microorganisms most frequently associated with outbreaks of vegetable-borne diseases being bacteria such as *Salmonella* spp., *Listeria monocytogenes*, *Clostridium botulinum*, *Escherichia coli*, and *Shigella* spp. [6]. In addition, due to their high water and nutrient content, minimally processed fruits and vegetables are highly vulnerable to microbial growth, as they lose their natural resistance [7]. According to various studies, most cases of food contamination in the United States between 2010 and 2017 were caused by *Salmonella enterica* and *Escherichia coli*, which were linked to the consumption of fresh vegetables [8]. The high percentage of infections caused by the consumption of these vegetables, according to the USDA (United States Department of Agriculture), was associated with the growing trend towards consuming green vegetables and packaged fresh-cut salads [9]. While food safety is a legitimate concern and many efforts are being made by various public health administrations around the world to reduce food contamination, there is still much work to be performed to ensure food safety in retail sales and in handling at the home and collective catering levels, as the number of foodborne outbreaks in 2023 increased significantly in Europe, with 5691 cases of food poisoning reported, of which “vegetables, juices and their derivatives” were the foods that contributed most to these food outbreaks compared to previous years [10]. Among these foods, stalked vegetables tend to have a higher microbial load than sessile vegetables, as the stalk is a functional part of the plant that transports water and nutrients and where pathogenic microorganisms capable of forming biofilms, such as *L. monocytogenes* or *E. coli O157:H7* (among others), remain attached even after disinfection [11]. In itself, the petiole is not inherently a source of bacterial contamination, but it can become a source of contamination if the plant suffers an infection, especially if it comes into contact with human or animal faecal matter [12]. This highlights the importance of properly disinfecting vegetables (especially those with petioles) before consumption, especially if they are to be eaten raw.

Several chemicals are used for disinfection in both domestic and industrial vegetable preparation, with sodium hypochlorite (chlorine) being one of the most widely used disinfectants due to its broad spectrum of action against a wide variety of bacteria and viruses, as well as its cost-effectiveness. However, recent studies have highlighted the health risks associated with the use of this chemical disinfectant. Sodium hypochlorite (a fundamental component of bleach solutions) reacts with the organic parts of food, producing undesirable by-products such as trihalomethanes, which are potentially carcinogenic [13]. Furthermore, it is still unclear whether these substances will end up in drains, thus contaminating water supplies and the environment. As a result, the use of sodium hypochlorite is currently under review by health authorities in several European countries, including Germany, the Netherlands, and Switzerland [14].

For these reasons, it is important to investigate natural, household-accessible methods for disinfecting vegetables, since conventional chemical methods, such as sodium hypochlorite, pose a risk to health and the environment. The study by Esmael et al., 2023, highlighted the need to investigate a new disinfectant alternative for vegetables to eliminate bacterial pathogens, while preserving functional value and contributing to human safety [15].

To achieve this purpose, many studies have investigated the use of other alternatives, including chlorine dioxide, ozone, organic acids, peroxyacetic acid, electrolyzed oxidising water, and hydrogen peroxide [16,17]. Essential oils have recently gained relevance as potential tools for food preservation and as antimicrobial agents [18], investigating their possible use as disinfectants against microorganisms such as *Escherichia coli* and *Staphylococcus aureus* [19].

On the other hand, antioxidants play a crucial role in maintaining our health by neutralising harmful free radicals, which can cause cell damage and contribute to various diseases, such as cancer, cardiovascular disease, and neurodegenerative disorders [20]. Vegetables are a rich source of antioxidants, providing a natural defence system for our body. Polyphenols, especially flavonoids and phenolic acids, which are abundant in green leafy vegetables such as spinach, kale, broccoli, and lettuce, help protect our cells from oxidative stress. The regular consumption of vegetables rich in these antioxidants promotes overall health, strengthens the immune system, and reduces the risk of chronic diseases, making them an essential component of a balanced diet [21]. In relation to essential oils, thyme essential oil has powerful antioxidant properties thanks to its high content of phenolic compounds, particularly carvacrol and thymol. These compounds effectively neutralise free radicals, protecting cells from oxidative damage. However, these antioxidant compounds, especially phenolic compounds, can undergo a process of oxidation, giving rise to highly reactive quinones that can react with the amino groups of proteins, forming complexes that are difficult to absorb in the intestine, which can reduce the availability of these nutrients. Furthermore, as they are highly reactive chemical species, they can generate oxidative stress and damage cells [22].

This study aims to evaluate the influence of thyme, peppermint, and bleach solution essential oils on radical scavenging activity and total phenolic content when used as disinfectants for baby spinach (petiolate vegetable) and *Romaine lettuce* (sessile vegetable). It also evaluates the relationship between microbial load, antiradical activity, and polyphenolic content in vegetables treated with these disinfection methods to determine whether essential oils can guarantee their disinfectant power and maintain or increase the radical scavenging activity of these vegetables and their comparative effectiveness with classic disinfectants such as bleach solutions.

## 2. Materials and Methods

### 2.1. Samples Used

The samples analysed were fresh stalked vegetables (baby spinach, n = 36) and fresh sessile vegetables (*Romaine lettuce*, n = 36). The samples were collected from local greengrocers in Spain, placed in sterile bags under aseptic conditions, and microbiologically analysed on the day of purchase. In the case of spinach, the leaves were cut and mixed with the stems. With regard to essential oils, thyme and mint oils (suitable for food consumption) purchased from local herbalists in opaque glass bottles were used, and chlorine bleach solutions labelled as “suitable for human consumption” were also purchased from local supermarkets in Spain. The rest of the reagents used are from Sigma-Aldrich^®^, Darmstadt, Germany.

### 2.2. Disinfection Treatments Used

The vegetable samples (baby spinach and *Romaine lettuce*) were disinfected with thyme and mint essential oils and bleach solutions. To do this, 10 g of each vegetable was immersed in 200 mL of water, adding the disinfectants in different concentrations. The vegetables were left to soak for 5 min for the disinfectant to take effect [23]. After this time, the samples treated with bleach were washed with running water until the pH of the wash water reached a value of 7 (to eliminate the risk of trihalomethane formation). This washing was not necessary for the samples treated with essential oil. A salad spinner was used to remove excess water from the leaves of all samples. Although there are no universal recommendations on the use of specific amounts of essential oil as a food disinfectant, several studies have determined that the no observed adverse effect level (NOAEL) for an essential oil is approximately 32.78 mg/kg/day [23,24]. In our study, we selected three concentrations significantly below this limit:

- ***Disinfection treatment A***: 200 mL of water + 0.04 mL of essential oil + 10 g vegetable.
- ***Disinfection treatment B***: 200 mL of water + 0.08 mL of essential oil +10 g vegetable.
- ***Disinfection treatment C***: 200 mL of water + 1.2 mL of essential oil +10 g vegetable.

In Spain, according to RD 3360/1983, chlorine bleach solutions suitable for disinfecting drinking water and vegetables must have an active chlorine content of between 35 g/L and 60 g/L. In our study, we used bleach solutions with an active chlorine content of 35 g/L, following the recommendations of the Catalan Food Safety Agency [25].

- ***Disinfection treatment D***: 200 mL of water + 0.08 mL bleach solution +10 g vegetable.
- ***Disinfection treatment E***: 200 mL of water + 0.2 mL bleach solution + 10 g vegetable.

### 2.3. Microbiological Analysis Methods

Microbial counts of mesophiles, psychrophiles, yeasts, and moulds were performed, as well as non-spore-forming microorganisms that can indicate the efficiency of production and disinfection processes. *Listeria monocytogenes* is a dangerous non-spore-forming pathogen, and its presence in vegetables is critical, as it indicates a risk of disease and the need to review disinfection protocols. Microbiological analyses were performed on both baby spinach and *Romaine lettuce* samples (before and after undergoing the different disinfection treatments). Sample preparation (for the microbial count, 1 g of each vegetable diluted in 9 mL of sterile buffered peptone water solution, and, from these, decimal dilutions were made) and microorganism enumeration procedures were performed using official techniques, namely ISO 4833:2003 standard for aerobic mesophilic and psychrophilic bacteria [26], ISO 21527-2:2008 for moulds and yeasts [27] and ISO 11290: 2017 for *L. monocytogenes* [28]. The results have been expressed as microbial count values (Log CFU/g) and standard error of the mean (SEM) and are the result of 10 determinations for each microorganism. The culture media and peptone water are from Merck®. 

### 2.4. Radical Scavenging Activity (RSA)

To determine the radical scavenging activity of both the baby spinach and *Romaine lettuce* samples, the 2,2-diphenyl-1-picrylhydrazyl radical neutralisation method described by [29] was used. The DPPH method was used because it best suited our experimental conditions and the samples analysed, according to the literature found [30]. Similarly, the extraction method described by Lavelli (2002) was chosen, with some modifications used [31]. The antiradical activity was determined by reading the absorbance at 516 nm in a Perkin Elmer Lambda 25 UV/VIS Spectrophotometer, and the following equation was used to measure the inhibition coefficient using methanol as a blank:

I% = (A_control_ − A_sample_/A_control_) × 100 (1)

(1) A_control_ and A_sample_ refer to the absorbance of the DPPH solution and the vegetable studied, respectively.

The results are expressed in μM Trolox/g sample and are the result of 10 determinations performed on each sample. To check for differences in RSA between vegetables, radical scavenging activity was evaluated in both untreated samples and samples treated with different treatments. The data are reported in the figures as “mean ± SD”.

### 2.5. Total Phenolic Compounds (TPC)

The total phenolic content was determined using the Folin–Ciocalteu spectrophotometric method [32] based on the reaction of phenolic compounds with the Folin–Ciocalteu reagent, forming an intense blue complex that is quantified by measuring the absorbance at a specific wavelength of 765 nm. The concentration was determined by comparing the absorbance of the sample with a calibration curve prepared with a reference standard, such as gallic acid. The regression equation obtained had a regression coefficient of R^2^ = 0.9747.

The concentration of phenols in vegetable samples (before and after undergoing the different disinfection treatments) was calculated using this calibration curve and expressed in milligrams of gallic acid equivalents (mg GAE) per gram of sample. Data are mentioned in figures as “mean ± SD” and are the result of 10 determinations performed on each sample.

### 2.6. Statistical Analysis

SPSS 28.00 for Windows (IBM SPSS Inc., New York, NY, USA) program was used for data analysis. The data were expressed as the mean and standard error of the mean based on 10 determinations per sample performed. ANOVA or the Kruskal–Wallis test was applied to the means and to determine whether statistically significant differences exist. Pearson’s and Spearman’s correlation coefficients were applied for normally and non-normally distributed data, respectively. Additionally, Pearson’s correlation coefficients were calculated to analyse the relationship between antioxidant activity/total phenolic content and microbial counts. The significance level set for the tests was 5% (*p* < 0.05). Furthermore, discrimination analysis was performed using Statgraphics (Statgraphics technologies 16, Inc., The Plains, VA, USA) to evaluate the ability of the studied parameters to distinguish between treatments groups and identify the most influential variables contributing to group separation.

## 3. Results and Discussion

### 3.1. Microbial Counts According to the Disinfection Treatment Used

The results of the microbial analyses carried out according to the disinfection system used and expressed as percentages of microbial reduction are shown in Table 1 (petiolate vegetable, baby spinach) and Table 2 (sessile vegetable, *Romaine lettuce*) and are the result of the average obtained after applying each disinfection treatment 10 times for each sample analysed. As expected, the disinfection treatments with thyme and peppermint essential oils and bleach solutions showed a progressive increase in the percentage of microbial reduction as the concentration of essential oils and aqueous sodium hypochlorite solutions (bleach solutions) increased.

The initial microbial load (before disinfection) in sessile vegetables was as follows: mesophilic microorganisms, Log CFU/g 5.14 ± 0.4; psychrophilic microorganisms, Log CFU/g 7.46 ± 0.9; moulds and yeasts, Log CFU/g 6.59 ± 0.3; and *Listeria monocytogenes*, Log CFU/g 5.81 ± 0.2. For petiolated vegetables, the microbial counts obtained were as follows: mesophilic microorganisms, Log CFU/g 5.98 ± 0.3; psychrophilic microorganisms, Log CFU/g 8.7 ± 0.9; moulds and yeasts, Log CFU/g 6.96 ± 0.2; and *Listeria monocytogenes*, Log CFU/g 6.11 ± 0.3.

Both oils achieved a reduction in the initial microbial load in young spinach following the same dynamics as in sessile vegetables, with peppermint oil (and treatment C) achieving the greatest reductions (*p* < 0.05). Bleach solutions produced the highest reduction percentages with treatment E, similar to essential oils, except in the case of mesophilic microorganisms (*p* < 0.05). In this study, thyme and peppermint essential oils significantly reduced (*p* < 0.05) the microbial load in baby spinach and *Romaine lettuce* samples for all microorganisms analysed and were highly effective against *Listeria monocytogenes*. This finding is consistent with the results described by other authors, who highlighted the high antimicrobial efficacy of thyme oil even at low concentrations, mainly attributed to the presence of thymol and carvacrol [33,34].

Similarly, previous studies have associated the presence of menthol and menthone in peppermint essential oil, with its protective effect against oxidative stress and its inhibitory action on pathogenic bacteria [35,36].

According to the findings of Bibow and Oleszek (2024), essential oils with a high antiradical activity, such as thyme and peppermint, have greater antimicrobial efficacy because the same polyphenolic compounds responsible for neutralising free radicals also generate a redox imbalance within microbial cells, weakening their defence mechanisms [37]. A similar study explains that the increase in radical scavenging activity of essential oils is associated with an increase in intercellular oxidative stress in microorganisms, which exceeds their endogenous antiradical activity and causes the loss of cell viability [38]. All these studies corroborate our findings, which show that the use of thyme and peppermint essential oils has a strong disinfectant effect on fresh vegetables, especially peppermint oil, with strong differences (*p* < 0.05) in the reduction in mesophilic microorganisms, psychrophilic microorganisms, moulds, yeasts, and *Listeria monocytogenes*. These microorganisms are used to evaluate the general hygiene conditions and the effectiveness of the disinfection and handling processes of these plant foods [11]. We also observed that this reduction in microbial load is generally greater in sessile vegetables (*Romaine lettuce*) than in petiolate vegetables (baby spinach) and could be due to the presence of the petiole, which acts as a natural reservoir for some of these food pathogens, which would be consistent with other authors [11].

In Spain, it is recommended to use bleach solutions to disinfect vegetables that are eaten raw [25]. In this study, we demonstrate that, although these bleach solutions have antimicrobial properties, there are no differences in disinfecting power between thyme and peppermint oils and these chemical disinfectants. Furthermore, these essential oils would be harmless to consumer health, whereas the chlorine in bleaching solutions reacts with the organic matter in vegetables, producing harmful by-products such as trihalomethanes (THMs), which means that a thorough rinsing with water is necessary to remove chlorine residues. In countries such as Spain, which is suffering from severe drought, this use of water is an unnecessary waste. These chlorine residues can remain on vegetables and be ingested, and in the long term have been associated with an increased risk of cancer [14,39].

### 3.2. Radical Scavenging Activity (RSA) in Petiolated Vegetables and Sessile Vegetables

**Figure 1:**
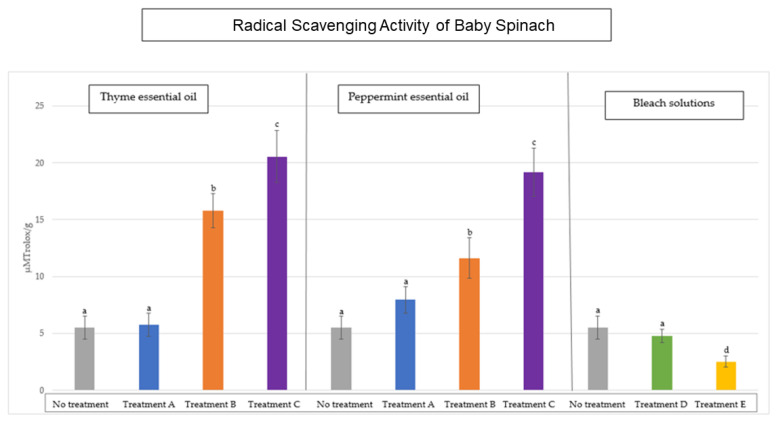
Values of radical scavenging activity (RSA) in petiolate vegetables (baby spinach) after decontamination with thyme oil, peppermint oil, and bleach solutions compared to the initial load without disinfection. Different letters indicate significant differences between the samples analysed (*p* < 0.05).

**Figure 2:**
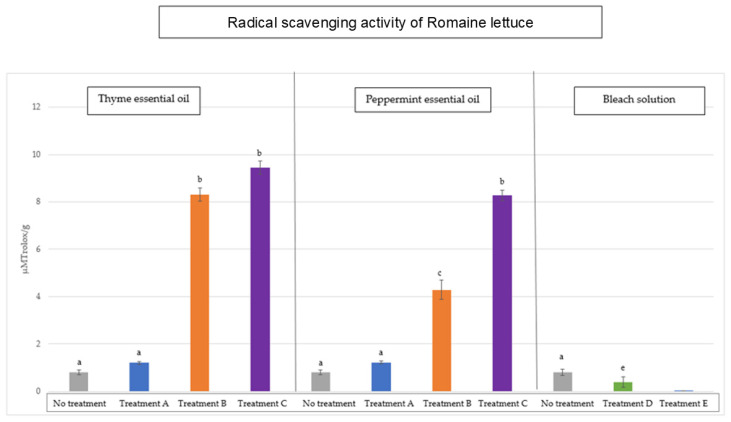
Values of radical scavenging activity (RSA) in sessile vegetables (*Romaine lettuce*) after decontamination with thyme oil, peppermint oil, and bleach solutions compared to the initial load without disinfection. Different letters indicate significant differences between the samples analysed (*p* < 0.05).

Figure 1 and Figure 2 show the values obtained for the radical scavenging activity in the baby spinach and *Romaine lettuce* samples, respectively, after the different disinfection systems applied. The initial mean values, before disinfection, for petiolated vegetables are 5.51 ± 0.3 μM Trolox/g sample and, for sessile vegetables, 0.79 ± 0.2 μM Trolox/g sample.

As can be seen in Figure 1, the RSA of baby spinach increased after disinfection with peppermint and thyme essential oils. If we compare the RSA value obtained after applying disinfection to these samples, we can see that there are no statistically significant differences between peppermint oil and thyme oil for treatment A. The same dynamic is established for the other two disinfection treatments applied (B and C). On the other hand, when we use bleach solutions, we see that the radical scavenging activity of these vegetables decreased, especially in treatment E (*p* < 0.05), as these bleach solutions have a high oxidising power which, although effective in destroying viruses and bacteria, can also alter or degrade the antioxidant compounds in vegetables. In the case of sessile samples, the effect on the antiradical activity (Figure 2) of these oils follows a similar dynamic.

Therefore, thyme and peppermint essential oils rich in phenolic compounds, especially thyme oil in carvacrol and thymol [40], when used as natural disinfectants for fresh vegetables, have a positive effect on radical scavenging activity, while significantly reducing the microbial load.

In relation to bleach solutions, a decrease (*p* < 0.05) in total antiradical activity was observed in all samples analysed. A study by Alimohammadi et al. (2016) investigated the effect of certain chemical disinfectants on radical scavenging activity in lettuce and concluded that the use of benzalkonium chloride causes a decrease in total antiradical activity in vegetables disinfected in this way [41]. Other studies have linked the use of chemical disinfectants, including sodium hypochlorite, to the creation of harmful by-products such as trihalomethanes (THMs), which are formed when organic matter in vegetables reacts with the chemical disinfectant [14,39]. These by-products are produced in oxidative reactions of organic matter and ultimately create oxidative stress, which could explain the decrease in available radical scavenging activity [42].

### 3.3. Total Phenolic Compounds (TPCs) in Leafy Vegetables and Sessile Vegetables

**Figure 3:**
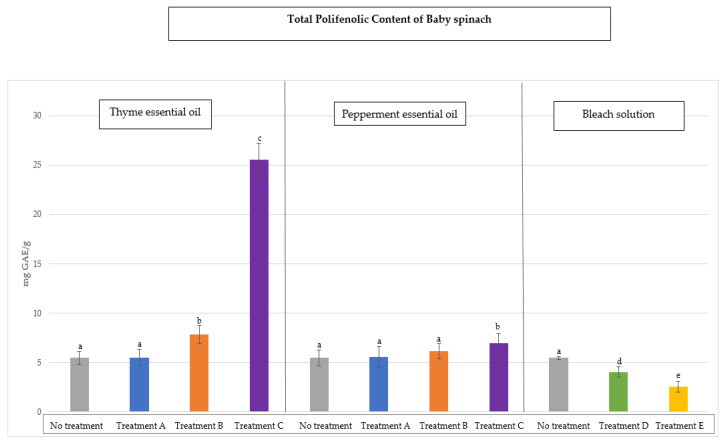
Values of total phenolic compounds (TPCs) in petiolate vegetables (baby spinach) after decontamination with thyme oil, peppermint oil, and bleach solutions compared to the initial load without disinfection. Different letters indicate significant differences between the samples analysed (*p* < 0.05).

**Figure 4:**
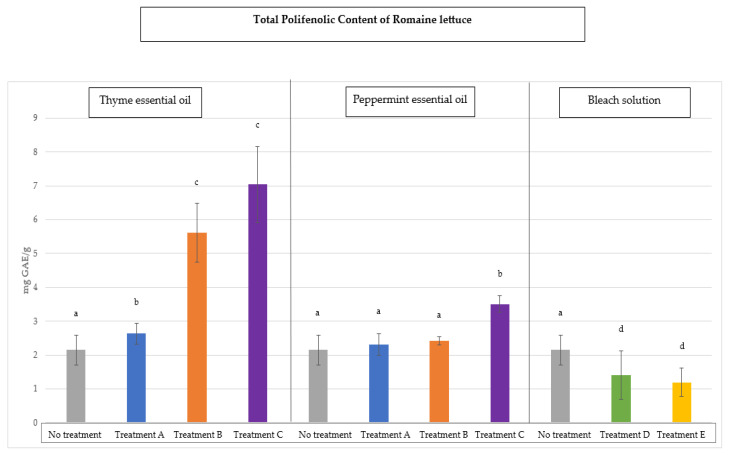
Values of total phenolic compounds (TPCs) in sessile vegetables (*Romaine lettuce*) after decontamination with thyme oil, peppermint oil, and bleach solutions compared to the initial load without disinfection. Different letters indicate significant differences between the samples analysed (*p* < 0.05).

The total phenolic compound (TPC) values for the baby spinach and *Romaine lettuce* samples are shown in Figure 3 and Figure 4, respectively. These values are expressed as milligrams of gallic acid equivalents (mg GAE) per gram of sample and represent the “mean ± SD” as a result of 10 determinations carried out on each sample and for each disinfection treatment followed. The value obtained for the baby spinach samples before disinfection was 5.46 ± 0.4 mg GAE/g sample and 2.14 ± 0.2 mg GAE/g for the sessile vegetable samples.

In all vegetables (Figure 3 and Figure 4), a significant increase in the total phenolic compounds (*p* < 0.05) was observed, especially when thyme oil was used in treatment C. Peppermint oil, although it slightly increased this TPC value compared to that of the non-disinfected vegetable samples, is not as significant. When bleach solutions were used, the total phenolic content decreased considerably (*p* < 0.05) compared to both essential oils. As expected, and as with radical scavenging activity, the disinfection with bleach solutions (due to their strong oxidising nature) reduced the total phenolic content in vegetable samples, demonstrating through these data how the oxidising power of bleach affected the natural antioxidants, causing a loss of total phenolic compounds.

These results are consistent with those of other authors [43], who demonstrated that the use of sodium hypochlorite as a disinfectant for fresh vegetables can have negative effects due to a significant accumulation of chlorates and other disinfection by-products with high oxidising power in these foods.

### 3.4. Relationship Between Radical Scavenging Activity, Microbial Load, and Total Phenolic Content

In view of these results and given that essential oils reduce the microbial load and increase the RSA and TPC of vegetables when used as disinfectants, while bleach disinfectant solutions, as they decrease the microbial load, also produce a significant decrease in both the RSA and TPC of vegetables, the Pearson correlation coefficient (Table 3) was applied to see if there is a linear relationship and proportionality between these variables according to the type of disinfectant applied, essential oils and bleach solutions (Tables S1–S4)

Thyme oil and peppermint oil showed a strong to very strong inverse correlation between antiradical activity and microbial load and TPC. This correlation value confirms that essential oils reduce microbial load while increasing RSA and TPC, demonstrating that disinfection with essential oils has a positive effect on vegetables. On the other hand, when bleach solutions were used, the direction of this correlation changed and became a very strong direct correlation, confirming the results obtained after the disinfection processes, as these bleach solutions not only reduced the microbial load but also the radical scavenging activity and TPC of the vegetables thus disinfected. In the case of vegetables with petioles (baby spinach), this inverse correlation between radical scavenging activity and microbial load and total phenolic compounds was lower than in sessile vegetables.

This relationship between the total increase in polyphenols and the reduction in microbial load can be explained by the same mechanisms that link antiradical activity to microbial load, as explained in the previous section. The polyphenolic compounds present in essential oils have a dual antioxidant and antimicrobial function. On the one hand, they act as free radical scavengers, reducing oxidative stress and protecting molecules from oxidation, and, on the other hand, their lipophilic nature interacts with the lipids of the microbial membrane, causing cell death [37,44,45]. Other authors [46], when applying essential oil coatings in the form of edible films to extend the shelf life of fruits such as grapes, found an improvement in the antioxidant status and polyphenolic content of these fruits, which can be explained by the induction of defence mechanisms in the grapes treated in this way. However, further studies are needed to analyse the changes in the phenolic profile of grapes treated with these edible coatings to support this claim. Almajano and Gordon [47] recognise the synergistic effect of γ- and other tocopherols (very abundant in thyme oil) in protecting β- and other carotenoids during oxidation reactions, which could explain the increase in RAS in the vegetables disinfected by us with these essential oils.

**Figure 5:**
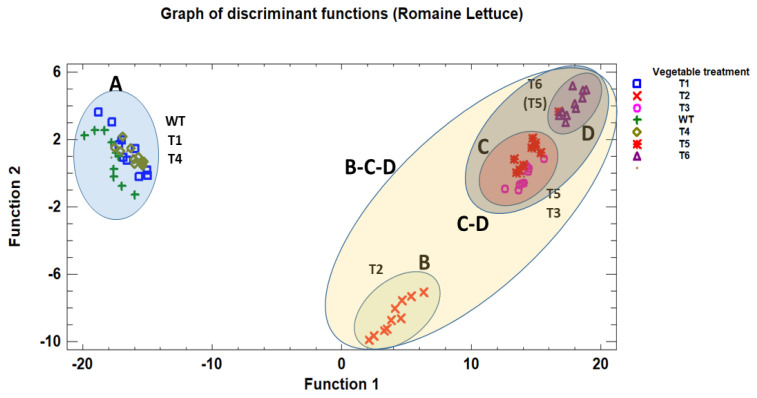
Discriminant analysis of different treatments based on essential oils applied to *Romaine lettuce* (sessile vegetable). Vegetable treatment: T1: peppermint_Treat.A; T2: peppermint_Treat.B; T3: peppermint_Treat.C; T4: thyme_Treat.A; T5: thyme_Treat.B; T6: thyme_Treat.C; WT: Without_Treatment.

**Figure 6:**
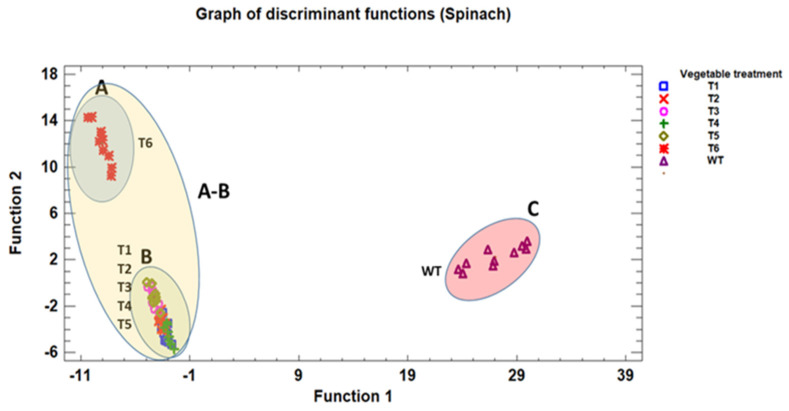
Discriminant analysis of different treatments based on essential oils applied to baby spinach (petiolate vegetable). Vegetable treatment: T1: peppermint_Treat.A; T2: peppermint_Treat.B; T3: peppermint_Treat.C; T4: thyme_Treat.A; T5: thyme_Treat.B; T6: thyme_Treat.C; WT: Without_Treatment.

As we have seen, the effect of essential oils differs depending on the type of vegetable (petiolate or sessile) and the type of essential oil. In order to identify significant differences between the two types of vegetables and the effect that the oils used in the disinfection process have on them, a discriminant analysis was carried out (Figure 5 and Figure 6).

The discriminant analysis applied to the *Romaine lettuce* samples (Figure 5) allowed the distribution of the treatments to be visualised according to the first two discriminant functions (Figure 6). The resulting plane clearly distinguishes several clusters, labelled A, B, C, and D, which exhibit different behaviours among the treatments applied. Group A, located on the left side of the graph, groups the untreated control together with the disinfection treatment A with thyme and mint essential oils. These samples are positioned very close to each other, showing similar behaviour within the discriminant space. Group B is located towards the lower centre of the graph and consists of samples corresponding to disinfection treatment B with peppermint essential oil. This group appears separate from the previous one along the first discriminant function. Groups C and D are located on the right side of the graph, grouping disinfection treatment C with thyme and peppermint essential oils and disinfection treatments D and E with sodium hypochlorite. These samples are distributed in two sub-areas close to each other, differentiated from the rest of the treatments and the untreated group.

Overall, the discriminant representation shows a clear separation between the samples on the far left (group A) and the clusters on the right (groups C and D), with an intermediate transition represented by group B. This distribution shows the existence of four main regions (A, B, C, and D) in the plane of discriminant functions, with well-defined positions and no overlap between the extreme groups.

The discriminant analysis of the different treatments applied to the spinach samples revealed a clear separation between the groups defined according to the first two discriminant functions (Figure 6).

Within the space formed by these functions, three main groups can be distinguished (A, B, and C). Group A consists of disinfection treatment C with thyme essential oil, located in the upper left part of the graph. This group is isolated from the rest, indicating a different behaviour compared to the other samples. Group B includes disinfection treatments A and B, applied with mint and thyme essential oils. These samples are located in the lower left of the graph, close to each other, showing a great similarity within the group. Group C corresponds to the untreated control, located separately on the far right of the discriminant plane. This group is clearly distinct from the others, with no overlap with regions A or B.

Comparing both vegetables, it can be observed that essential oils do not act uniformly in the disinfection process, nor on the RSA and TPC values of the vegetables. Although the disinfection treatment applied is discriminatory, it is the type of vegetable that marks the differences. In *Romaine lettuce*, thyme oil showed a greater ability to modify microbiological counts depending on the disinfection treatment used, demonstrating a more direct action on the leaf surface. In contrast, in spinach with petioles, peppermint oil stood out for its more pronounced effect on antiradical activity and polyphenolic compound content, especially for treatments B and C, but with less reducing power against microorganisms. This could be because the structure of the spinach leaf, with a more developed petiole, facilitates the adhesion of biofilm-forming microorganisms [11] and increases their resistance to disinfection, while its leaves with a larger leaf surface facilitate the absorption of the active compounds in mint, enhancing its antioxidant effect.

In general, the differential behaviour of the oils between the two vegetables shows that the effectiveness of essential oils depends both on their chemical composition and on the morphological characteristics of the treated vegetable. Thyme tends to show a more stable and predominantly antimicrobial effect on sessile vegetables (*Romaine lettuce*) depending on the treatment applied, which would be consistent with the results reported by other authors [45], while peppermint has a more variable action but with a greater impact on antioxidant capacity and TPC in petiolate vegetables [14,15].

## 4. Conclusions

The polyphenols found in thyme and mint essential oils have a dual action as antioxidants and antimicrobial agents. As antioxidants, they neutralise the free radicals that cause oxidative stress, increasing the radical scavenging activity (RSA) and total phenolic content (TPC) of vegetables disinfected by them, while bleach solutions, due to their strong oxidising character, produce a significant decrease in both the RSA and TPC. As antimicrobials, their lipophilic nature allows them to interact with the lipids in the cell membrane of microbes, causing their death. This antimicrobial and antioxidant effect depends on the morphological characteristics of the vegetable (stalked or sessile) and the chemical composition of the essential oil. While peppermint oil has a more pronounced effect as an antimicrobial agent in sessile vegetables (*Romaine lettuce*), thyme oil has a greater impact on the antiradical activity and total phenol content in petiolate vegetables (baby spinach). Compared to bleach solutions, both oils show a reduction in microbial load similar to disinfectant bleach but without the adverse effects of the latter, making them promising disinfectants of choice for fresh vegetables.

## Figures and Tables

**Table 1:** Microbial reduction (%) in sessile vegetables (*Romaine lettuce*) after decontamination with thyme oil, peppermint oil, and bleach solutions. Different letters within the same column indicate significant differences between the samples analysed (*p* < 0.05).

Thyme Essential Oil				
Decontamination Treatment	Mesophilic Microorganisms	Psychrophilic Microorganisms	Moulds and Yeasts	*L. monocytogenes*
Treatment A	37.40 ^a^	75.40 ^a^	72.20 ^a^	82.00 ^a^
Treatment B	81.40 ^b^	96.00 ^b^	91.70 ^b^	96.55 ^b^
Treatment C	91.30 ^c^	96.70 ^b^	93.90 ^b^	100.00 ^c^
**Peppermint Essential Oil**				
Decontamination Treatment	Mesophilic Microorganisms	Psychrophilic Microorganisms	Moulds and Yeasts	*L. monocytogenes*
Treatment A	28.70 ^a^	96.30 ^b^	77.40 ^a^	94.60 ^b^
Treatment B	91.30 ^c^	98.30 ^c^	98.50 ^c^	97.70 ^b^
Treatment C	92.80 ^c^	98.60 ^c^	99.70 ^c^	100.00 ^c^
**Bleach Solution**				
Decontamination Treatment	Mesophilic Microorganisms	Psychrophilic Microorganisms	Moulds and Yeasts	*L. monocytogenes*
Treatment D	90.80 ^c^	98.50 ^c^	96.20 ^c^	100.00 ^c^
Treatment E	96.90 ^d^	98.90 ^c^	97.80 ^c^	100.00 ^c^

**Table 2:** Microbial reduction (%) in petiolate vegetables (baby spinach) after decontamination with thyme oil, peppermint oil, and bleach solutions. Different letters within the same column indicate significant differences between the samples analysed (*p* < 0.05).

Thyme Essential Oil				
Decontamination Treatment	Mesophilic Microorganisms	Psychrophilic Microorganisms	Moulds and Yeasts	*L. monocytogenes*
Treatment A	38.60 ^a^	81.00 ^a^	13.10 ^a^	37.90 ^a^
Treatment B	67.10 ^b^	82.80 ^a^	81.60 ^b^	100.00 ^b^
Treatment C	74.30 ^b^	88.70 ^a^	83.70 ^b^	100.00 ^b^
**Peppermint Essential Oil**				
Decontamination Treatment	Mesophilic Microorganisms	Psychrophilic Microorganisms	Moulds and Yeasts	*L. monocytogenes*
Treatment A	63.00 ^b^	79.70 ^b^	59.10 ^a^	37.90 ^a^
Treatment B	70.70 ^b^	85.30 ^a^	95.20 ^c^	100.00 ^b^
Treatment C	92.00 ^c^	91.60 ^c^	97.20 ^c^	100.00 ^b^
**Bleach Solutions**				
Decontamination Treatment	Mesophilic Microorganisms	Psychrophilic Microorganisms	Moulds and Yeasts	*L. monocytogenes*
Treatment D	23.60 ^a^	98.30 ^c^	89.40 ^b^	100.00 ^b^
Treatment E	58.60 ^b^	98.70 ^c^	95.50 ^c^	100.00 ^b^

**Table 3:** Correlation coefficients of radical scavenging activity versus microbial load and total phenolic compounds according to the disinfection systems used. ^a^ *p* < 0.05.

*Romaine lettuce* (Sessile Vegetable)					
	Mesophiles	Psychrophiles	Moulds and Yeasts	*Listeria*	TPC
Thyme Oil	−0.4159 ^a^	−0.4608 ^a^	−0.7336 ^a^	−0.9886 ^a^	0.8615 ^a^
Peppermint Oil	−0.6590 ^a^	−0.5252 ^a^	−0.7741 ^a^	−0.8717 ^a^	0.7667 ^a^
Bleach sol.	0.2332	0.6070 ^a^	0.6875 ^a^	0.8074 ^a^	0.4531 ^a^
**Baby Spinach (Petiolate Vegetable)**					
	Mesophiles	Psychrophiles	Moulds and Yeasts	*Listeria*	TPC
Thyme Oil	−0.5201 ^a^	−0.5877 ^a^	−0.6290 ^a^	−0.5541 ^a^	0.8068 ^a^
Peppermint Oil	−0.3656 ^a^	−0.4987 ^a^	−0.6611 ^a^	−0.5983 ^a^	0.5531 ^a^
Bleach sol.	0.4259 ^a^	0.3846 ^a^	0.5052 ^a^	0.5782 ^a^	0.7046 ^a^

## Data Availability

All data that support the findings of this study are available from the corresponding author upon reasonable request.

## Supplementary Materials

The following supporting information can be downloaded at https://www.mdpi.com/article/10.3390/antiox14121458/s1. Table S1: Antiradical activity and its correlation with microbial load in the petiolate vegetable (spinach). The correlation coefficient is considered weak when the r value is less than 0.4; moderate when the r value is between 0.5 and 0.7; and strong when the r value is greater than 0.7. Table S2: Antiradical activity and its correlation with microbial load in the sessile vegetable (*Romaine lettuce*). The correlation coefficient is considered weak when the r value is less than 0.4; moderate when the r value is between 0.5 and 0.7; and strong when the r value is greater than 0.7. Table S3: Total polyphenol compounds and their correlation with microbial load in the petiolate vegetable (spinach). The correlation coefficient is considered weak when the r value is less than 0.4; moderate when the r value is between 0.5 and 0.7; and strong when the r value is greater than 0.7. Table S4: Total polyphenol compounds and their correlation with microbial load in the sessile vegetable (*Romaine lettuce*). The correlation coefficient is considered weak when the r value is less than 0.4; moderate when the r value is between 0.5 and 0.7; and strong when the r value is greater than 0.7.
