# Supplementary material for: The Natural Disinfectant Role of Essential Oils in Improving Radical Scavenging Activity and Total Phenolic Compounds in Fresh Vegetables
Source: Antioxidants (Basel). 2025 Dec 5;14(12):1458. doi: 10.3390/antiox14121458 (PMC12729650; doi:10.3390/antiox14121458)
Supplement: Supplementary file 1 [file antioxidants-14-01458-s001.zip › antioxidants-3997092-supplementary.pdf]

Table S1: Radical scavenging activity and its correlation with microbial load in the petiolate vegetable (spinach). The correlation coefficient is considered weak when the r value is less than 0.4; moderate when the r value is between 0.5 and 0.7; and strong when the r value is greater than 0.7

Microorganism	Regression equation	Regression coefficient R^2	p
	**Thyme**		
**Mesophilic microorganisms**	y=-8,7948x+58,226	0,993	<0,01
**Psychrophilic microorganisms**	y=-9,5768x+70,608	0,862	<0,01
**Molds and yeasts**	y=-9,1394x+75,036	0,978	<0,01
Listeria monocytogenes	y=-1,3971x+14,048	0,570	0,053
	**Peppermint**		
**Mesophilic microorganisms**	y=-7,4247x+48,565	0,801	0,002
**Psychrophilic microorganisms**	y=-7,4918x+57,125	0,905	<0,01
**Molds and yeasts**	y=-6,8309x+57,714	0,996	<0,01
Listeria monocytogenes	y=-1,214x+12,928	0,622	<0,05
	**Sodium hypochlorite**		
**Mesophilic microorganisms**	y=-2,0953x-7,1674	0,876	0,002
**Psychrophilic microorganisms**	y=-1,8464x-7,3699	0,729	0,026
**Molds and yeasts**	y=-1,5122x-6,3888	0,780	0,013
Listeria monocytogenes	y=-0,4698x+2,6336	0,688	0,04

Table S2: Radical scavenging activity and its correlation with microbial load in the sessile vegetable (romaine lettuce). The correlation coefficient is considered weak when the r value is less than 0.4; moderate when the r value is between 0.5 and 0.7; and strong when the r value is greater than 0.7

Microorganism	Regression equation	Regression coefficient R^2	p
	**Thyme**		
**Mesophilic microorganisms**	y=-9,2287x+55,246	0,955	<0,001
**Psychrophilic microorganisms**	y=-7,6656x+49,732	0,726	0,008
**Molds and yeasts**	y=-6,1683x+45,943	0,972	<0,001
Listeria monocytogenes	y=-1,3679x+8,8564	0,995	<0,001
	**Peppermint**		
**Mesophilic microorganisms**	y=-5,2578x+32,006	0,932	<0,001
**Psychrophilic microorganisms**	y=-2,9441x+20,498	0,802	0,002
**Molds and yeasts**	y=-2,8147x+22,223	0,925	<0,001
Listeria monocytogenes	y=-1,2325x+8,3955	0,881	<0,001
	**Sodium hypochlorite**		
**Mesophilic microorganisms**	y=-2,0953x-7,1674	0,970	<0,001
**Psychrophilic microorganisms**	y=-1,8464x-7,3699	0,903	<0,001
**Molds and yeasts**	y=-1,5122x-6,3888	0,973	<0,001
Listeria monocytogenes	y=-0,4698x+2,6336	0,878	0,02

Tabla S3: Total polyphenol compounds and their correlation with microbial load in the petiolate vegetable (spinach). The correlation coefficient is considered weak when the r value is less than 0.4; moderate when the r value is between 0.5 and 0.7; and strong when the r value is greater than 0.7.

Microorganism	Regression equation	Regression coefficient R^2	p
	**Thyme**		
**Mesophilic microorganisms**	y=-10,226x+64,935	0,894	<0,01
**Psychrophilic microorganisms**	y=-10,223x+73,802	0,636	0,02
**Molds and yeasts**	y=-8,724x+71,341	0,809	<0,01
Listeria monocytogenes	y=-1,2267x+12,961	0,387	0,214
	**Peppermint**		
**Mesophilic microorganisms**	y=-5,2578x+32,006	0,760	0,004
**Psychrophilic microorganisms**	y=-2,9441x+20,498	0,885	<0,01
**Molds and yeasts**	y=-2,8147x+22,223	0,3727	<0,01
Listeria monocytogenes	y=-1,2325x+8,3955	0,564	0,056
	**Sodium hypochlorite**		
**Mesophilic microorganisms**	y=-2,0953x-7,1674	0,972	<0,01
**Psychrophilic microorganisms**	y=-1,8464x-7,3699	0,884	<0,01
**Molds and yeasts**	y=-1,5122x-6,3888	0,918	<0,01
Listeria monocytogenes	y=-0,4698x+2,6336	0,856	0,03

Tabla S4: Total polyphenol compounds and their correlation with microbial load in the sessile vegetable (romaine lettuce). The correlation coefficient is considered weak when the r value is less than 0.4; moderate when the r value is between 0.5 and 0.7; and strong when the r value is greater than 0.7.

Microorganism	Ecuación de regresión	Regression coefficient R^2	P
	**Thyme**		
**Mesophilic microorganisms**	y=-10,226x+64,935	0,967	<0,01
**Psychrophilic microorganisms**	y=-10,233x+73,802	0,766	0,04
**Molds and yeasts**	y=-8,724x+71,341	0,995	<0,01
Listeria monocytogenes	y=-1,2267x+12,961	0,1497	<0,01
	**Peppermint**		
**Mesophilic microorganisms**	y=-5,2578x+32,006	0,933	<0,01
**Psychrophilic microorganisms**	y=-2,9441x+20,498	0,743	0,06
**Molds and yeasts**	y=-2,8147x+22,223	0,780	0,03
Listeria monocytogenes	y=-1,2325x+8,3955	0,684	0,014
	**Sodium hypochlorite**		
**Mesophilic microorganisms**	y=-2,0953x-7,1674	0,762	0,017
**Psychrophilic microorganisms**	y=-1,8464x-7,3699	0,8422	<0,01
**Molds and yeasts**	y=-1,5122x-6,3888	0,991	<0,01
Listeria monocytogenes	y=-0,4698x+2,6336	0,7319	<0,01
